# Stent placement in pancreatic disease, when, which and why? – a current perspective

**DOI:** 10.3389/fgstr.2022.1039649

**Published:** 2023-01-16

**Authors:** Claudio C. Conrad, Mark Ellrichmann

**Affiliations:** Interdisciplinary Endoscopy, Department of Internal Medicine I, University Hospital Schleswig-Holstein, Kiel, Germany

**Keywords:** interventional endoscopy, pancreatic stenting, post-ERCP pancreatitis, pancreatic fistulae, pancreas divisum

## Abstract

**Introduction:**

Stenting of the pancreas is a challenging task for the interventional gastroenterologist. The indications for pancreatic stent implantation are either prophylactic or therapeutic. We give an overview of currently available literature and techniques for the respective indications of pancreatic stent placement.

**Methods:**

A structured literature research was conducted (Pubmed.gov) primarily using the following key words: interventional endoscopy, pancreatic stenting, post-ERCP pancreatitis, pancreatic Q8 fistulae, pancreas divisum.

**Results:**

Prophylactic stent implantation aims to prevent PEP by using thin (3-5 Fr) and short (3-5 cm) designated pancreatic stents at least in high-risk patients. Therapeutic stent placement is intended to restore the proper flow of pancreatic secretion with stenoses, leaks, fistulas or anatomical malformation of the pancreatic duct. Depending on the etiology, plastic stents or SEMSs are used. Another field of pancreatic stenting represents EUS-guided puncture with stent implantation as an alternative access to the main pancreatic duct when transpapillary access is impossible. In addition to the implantation of plastic stents, which achieve good results, LAMS implantation can be discussed as an alternative access route.

**Discussion:**

The field of pancreatic stenting is complex and belongs in the hands of experienced endoscopists in specialized institutions. This can ensure that the patient receives the optimal treatment with the best possible outcome.

## Introduction

The use of pancreatic stents is a major challenge in clinical routine due to the complex indications, potential complications and a great variety of different stent types. Therefore, it is important to choose the correct stent in the appropriate indication with balancing benefit and risks for the patient.

As an endoscopist, one should be well versed in the etiology, diagnostic and therapeutic techniques to develop an appropriate approach for each scenario.

In principle, the use of pancreatic stents can be divided into two areas of application: (i) prophylactic stenting and (ii) therapeutic stenting in benign or malignant indications. This article aims to provide a brief overview of the practical approach and to facilitate the selection of the right stent for the appropriate setting by answering the questions when, which and why.

## Indications for pancreatic stenting

### Prophylactic pancreatic stenting

When performing ERCP, post-ERCP pancreatitis (PEP) is a feared complication with a heterogeneously reported risk ranging between 2.1% and 24.4% in low/moderate risk patients and up to 40% in high-risk patients (1, 2). In a retrospective analysis the frequency of mild, moderate and severe PEP is reported to be 69%, 23%, and 8%, respectively (3).

According to the ESGE guideline the risk of PEP can be stratified in definite and likely risk factors as well as patient and procedure related risk factors. Definite patient-associated risk factors include a known sphincter Oddi dysfunction, female gender, and previous history of pancreatitis. Definite procedure-related risk for PEP exists with cannulation attempts lasting longer than 10 minutes, more than one guidewire passage of the pancreas, and contrast injection into the pancreatic duct (4) as summarized in **Table 1**. Of note, female patients with normal serum bilirubin level, suspected sphincter Oddi dysfunction and difficult cannulation encounter the highest risk of PEP of up to 42% (5).

**Table 1:** Risk stratification of post ERCP pancreatitis according to “Prophylaxis of post-ERCP pancreatitis: European Society of Gastrointestinal Endoscopy (ESGE) Guideline - updated June 2014” [8].

Patient-related risk factors	Procedure-related risk factors
*Definite*	
Suspected sphincter of Oddi dysfunction	Cannulation attempts duration >10 minutes
Female gender	Pancreatic guidewire passages >1
Previous pancreatitis	Pancreatic injection
*Likely*	
Previous PEP	Precut sphincterotomy
Younger age	Pancreatic sphincterotomy
Nondilated extrahepatic bile ducts	Biliary balloon sphincter dilation
Absence of chronic pancreatitis	Failure to clear bile duct stones
Normal serum bilirubin	Intraductal ultrasound

In any case, the ESGE recommends a rectal administration of 100 mg diclofenac or indomethacin periinterventionally if there are no known contraindications (i.e. reported allergies to NSAID) (4).

Meta-analyses of randomized-controlled trials have demonstrated a significant reduction of mild, moderate, and severe pancreatitis in high-risk patients with prophylactic stent placement (6, 7). Therefore, prophylactic stenting of the pancreas should be performed in the described high-risk constellations. However, it is important to note that the results are based on studies in which prophylactic non-steroid-anti-inflammatory-drugs (NSAIDs) were not routinely used.

**Figure 1:**
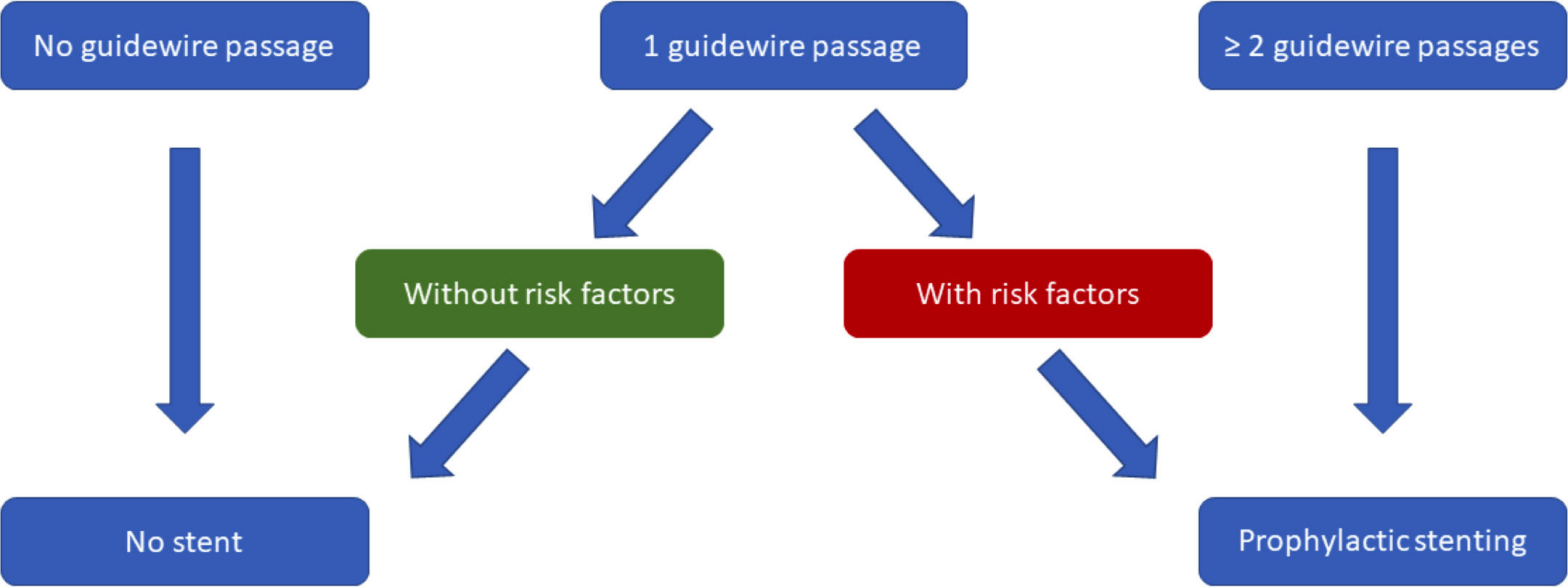
Suggested, simplified algorithm for prophylactic pancreatic stenting based on “Prophylaxis of post-ERCP pancreatitis: European Society of Gastrointestinal Endoscopy (ESGE) Guideline - updated June 2014” (8).

The stents used for this purpose should have an outer diameter of 3-5 Fr and a length of 3.5 cm to prevent additional damage to the duct system and in addition should have an external flap or pigtail to prevent migration (8). Stents should be removed after 5-10 days after insertion, though spontaneous migration into the duodenal lumen is observed in more than 95% within 2 weeks (9). For prophylactic stenting, we propose a simplified algorithm (**Figure 1**). Similarly, the use of a biodegradable stent could be considered making a following endoscopy for removal unnecessary.

Prophylactic stenting of the pancreatic duct also plays a prominent role in endoscopic papillectomy. Several studies have shown that the placement of a stent can significantly reduce the PEP rate (10, 11). A meta-analysis was able to demonstrate PEP reduction through stent implantation and described a possible risk reduction of late post-procedure papillary stenosis (12). In contrast to “pure” prophylactic pancreatic stenting, thicker (5 Fr) and longer (7cm) pancreatic stents should be preferred after papillectomy (8, 13).

## Therapeutic pancreatic stenting

### Pancreatic strictures

Strictures of the pancreatic duct exhibit major challenges to interventional endoscopists. Knowledge of the etiology of the stenosis (benign vs. malignant) is of utmost importance to select the appropriate treatment option and stent in the individual patient. Ruling out an underlying malignancy must an essential first step in the evaluation of PD strictures (14). Besides cross-sectional imaging technologies (CT scan, MRI/MRCP) and endoscopic ultrasound (EUS), direct sampling of the suspicious stricture can be obtained comprising EUS-guided FNA or FNB (fine needle aspiration cytology or fine needle biopsy under guidance of endoscopic ultrasound), fluoroscopically assisted endoscopic brush cytology and endoscopic biopsy and also direct pancreatoscopy with direct biopsy under visual control (15).

Benign pancreatic strictures can be further classified into simple/multiple and dominant/non-dominant (16). The most common causes of benign strictures are chronic pancreatitis (cP) and recurrent acute pancreatitis. Malignant strictures most often occur in the pancreas itself as pancreatic ductal adenocarcinomas, (lymph node-) metastases are the second leading causes of malignant obstructions. Dominant strictures are characterized by dilatation of the pancreatic duct to more than 6 mm proximal to the stenosis (17).

The endoscopic approach is the treatment of choice for symptomatic pancreatic strictures and lower rates of morbidity and mortality as compared to surgery (18). After pancreatic sphincterotomy, the stricture is identified by contrast imaging of the pancreatic duct. This is usually followed by dilatation of the corresponding section and stent implantation. This should be done in any case, as decompression by dilatation alone is not effective due to the tightness and elasticity of the strictures (19).

The most common cause of benign main pancreatic duct strictures (MPDS) is chronic pancreatitis with a prevalence of 50/100000 and abdominal pain being the leading symptom in 75% of patients at initial presentation (20). The primary goal of endoscopic intervention is therefore to reduce pain. As these are chronic changes, a cure cannot be achieved.

**Figure 2:**
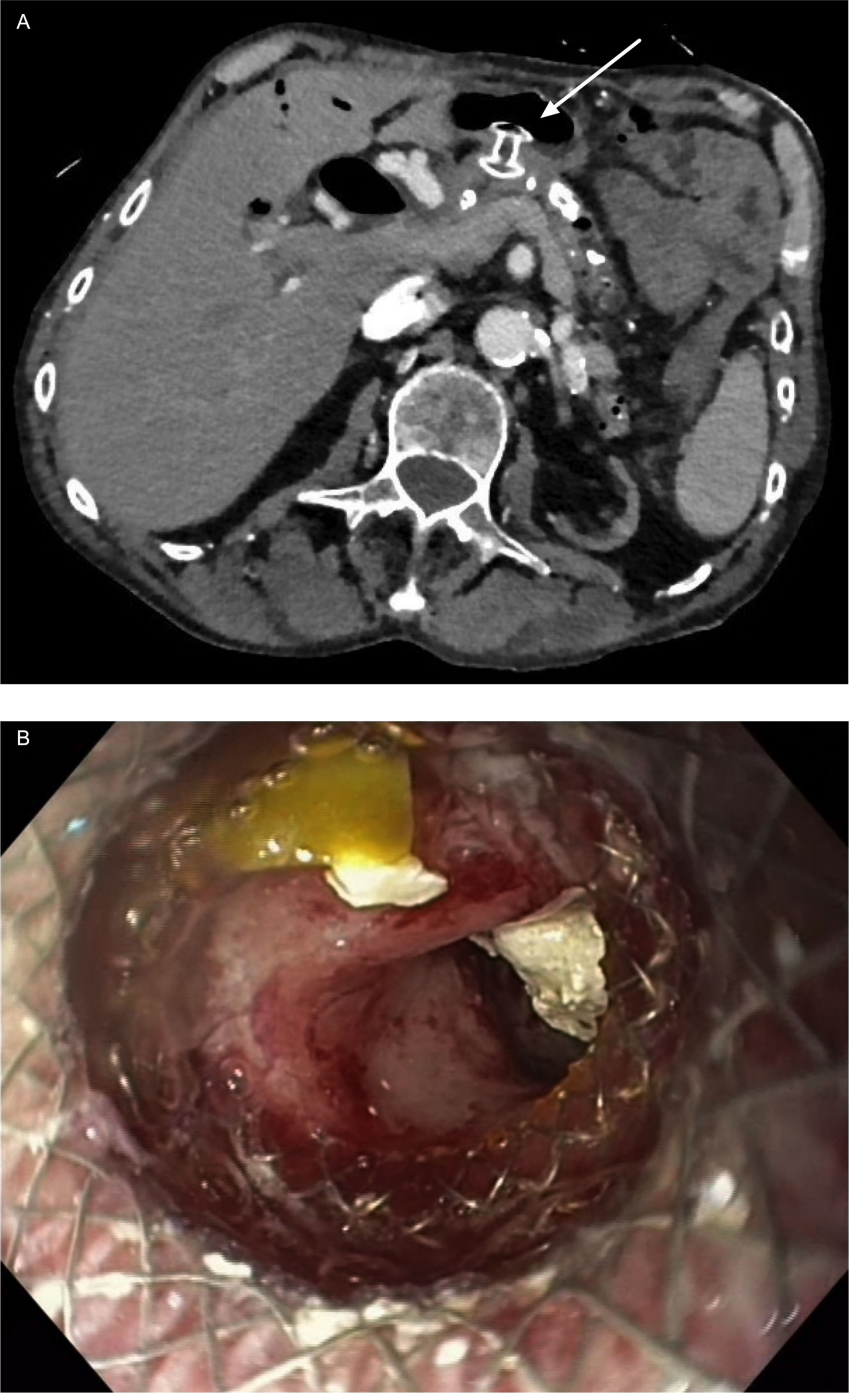
Case presentation of LAMS as gastro-pancreatostomy (HotAxios 8x8 mm, Boston Scientific, Marlborough, MA, USA): **(A)** CT scan showing LAMS in place in position of gastro-pancreatostomy; **(B)** transgastric view through LAMS into the pancreatic duct with residual stones fragments in the duct. (Pictures: Ellrichmann, Conrad).

The guidelines of American Society of Gastrointestinal Endoscopy (ASGE) and European Society of Gastrointestinal Endoscopy (ESGE) recommend plastic stent placement (16, 17), which results in an immediate clinical success rate of 83-100% and a long-term success rate of 84%, as measured by patients being relieved of pain (21–26). Both guidelines recommend implantation of a therapeutic 10 Fr diameter PPS across the dominant or most proximal stricture (**Figure 2**). Smaller stents tend to occlude more rapidly and subsequently result in a higher pain-related hospitalization rate (17, 19). For an effective treatment of stenosis, a median of 3 stent replacements is required in intervals of approximately 12 weeks (24).

ESGE suggests treatment with multiple side-by-side plastic stents as another option for refractory strictures defined by persistent or recurrent symptoms after one year of stent treatment. That this approach can also be successful has been demonstrated by a multicenter study in which 74.4% (32/43) patients were asymptomatic after stent removal at a mean follow-up of 9.5 years after multiple plastic stenting for stenoses in cP (22).

As an alternative to plastic stents, a self-expandable metal stent (SEMS) can be inserted. In the case of benign indications, this should always be fully covered (fcSEMS) to allow easy removal or replacement (27). A meta-analysis with 163 patients included could show a weighted pooled rate (WPR) of MPDS resolution of 93% (28). Furthermore, 78.5% of patients achieved persistent painlessness during a mean follow-up of 19.3 months (1-80 months) after stent removal. It could also be shown that a recumbency of up to 3 months compared to more than 3 months leads to a higher stricture recurrence (5% vs. 3%). The stents used were between 8 and 12 mm in diameter depending on the stricture and duct anatomy (27, 29). A comparative study showed that in direct comparison to multiple plastic stents, the clinical outcome in terms of pain improvement was similar (WPR 88% vs. 89%). However, the rate of adverse events was significantly higher in the SEMS group compare to the PPS group (WPR 38.6% vs. 14.3%) (30).

A new, currently still experimental, therapeutic approach for benign stenoses is the use of a biodegradable stent. So far, there is not much data on the use in pancreatic strictures. In a small case series 19 patients with MPDS were treated with a BDS (31). Inclusion criteria included confirmed cP as well as plastic stent insertion for at least 6 months without any sign of stricture resolution. The stent used was 6 mm in diameter and 3 or 4 cm in length, which limited treatment to stenoses in the pancreatic head. One-year follow-up showed a clinical success rate of 52% (11/19) with a procedure- and stent-related adverse event rate of 21% (4/19) (31). The results are promising, but the stent is not yet established for routine clinical use because further studies, especially on long-term outcomes, are still lacking. In addition, the stent that was used in the study is no longer available on the market.

Alternative BDS designed for stenosis that degrades within 11 weeks are available, but only with a maximum diameter of 3.4 mm.

For benign strictures, ASGE and ESGE clearly recommend the use of (multiple) plastic stents (16, 17). With a lower complication rate and lower costs, these are preferable to SEMSs or BDSs with a comparable outcome.

Malignant strictures of the pancreas are technically treated analogously to benign stenoses though they are usually treated in a palliative setting (if at all). Indication for drainage of malignant pancreatic strictures is rare and should only be considered if proper symptom control cannot be achieved by pain medication or celiac plexus neurolysis.

Both plastic and metal stents are used, depending on prognosis and location. Regarding diameter and length, the orientation is analogous to the benign indications. If a change is planned (e.g., if life expectancy is expected to be long), in our experience a scheduled change is preferable to an on-demand change, as this allows the individual progress of the local findings to be better addressed.

### Pancreatic leaks/fistulae

Pancreatic leaks and fistulas can occur in chronic or acute pancreatitis and after trauma to the pancreas or abdominal, especially pancreatic surgery. The maximum variant of a ductal defect is the disconnected or disrupted duct syndrome, in which the continuity of the pancreatic duct is completely lost.

Diagnosis is made by transabdominal and/or endoscopic ultrasound, computed tomography (CT), or secretin-enhanced magnetic resonance pancreatography (S-MRP) (32). Leaks and fistulas of the pancreatic tail play a special role. These are difficult to detect by imaging techniques due to their usually small size. In this case, contrast imaging of the pancreatic duct is the method of choice. Care must be taken to ensure that the contrast medium is injected in the tail region and not at papillary level (32).

While minor leaks often resolve with conservative management, major leaks regularly require intervention. As a first interventional step, EUS-guided drainage of the pancreatic fluid collection should be performed. If the collection does not resolve properly, endoscopic stenting of the corresponding section should be discussed for the treatment of leaks, fistulas or disconnected duct syndrome (DPDS). The aim is to guide the flow of pancreatic secretion into the duodenum and relieve the pressure from the leakage to allow healing of the corresponding section. The chances of success are between 77% for DPDS and 94% in fistulas (33, 34).

When selecting the stent, care must be taken to ensure that it bridges the leak adequately and does not have any lateral drainage holes. Therefore, plastic bile duct stents with a diameter of 5 to 7 Fr are used in this indication (34).

The shape of the stent should be adapted to the corresponding anatomical conditions. Straight stents with two flaps or single-pigtail stents with one flap can ensure adequate drainage with a low risk of migration. An alternative option is to use a BDS with a slow degrading profile (details see below).

There are no clear statements in the literature regarding a recommended duration of placement. In a small case series of 8 patients with postoperative pancreatic fistula, the median length of implantation was 48 days (35). We propose a re-evaluation with changing or removing the stent in a standard interval of 10-12 weeks depending on the clinical resolution of the leak.

### Pancreas divisum

Pancreas divisum (PD) is the most common anatomic variant of the pancreatic duct that affects 5-10% of all people (36). Most patients (more than 95%) are asymptomatic and do not require any treatment (37). The symptomatic patients, based on the most common clinical presentations, are divided into three subgroups of recurrent acute pancreatitis (RAP), chronic pancreatitis, and patients with chronic pain (38). Pathogenetically, dorsal ductal hypertension due to inadequate flow through a narrowed segment of the duct of Santorini and/or the papilla minor is thought to lead to recurrent acute and/or cP (39). However, it has been shown that most patients with a dilated dorsal duct do not show abdominal symptoms and the frequency of pancreatitis is similar compared to patients with normal duct variants (40, 41). In addition, several studies have shown that the prevalence of RAP and cP in children and adults with PD is up to 50% when certain genetic mutations of the serine protease inhibitor Kazal type 1 gene (SPINK1), cystic fibrosis transmembrane conductance regulator gene (CFTR) or chymotrypsin C gene (CTRC) are present (41–44). Therefore, it can be assumed that PD is a co-factor which, in interaction with other factors, leads to the pancreatic pathology.

Nevertheless, endoscopic treatment is the method of choice when dealing with a symptomatic PD (36–38, 45). The aim is the prevention of acute relapse and reduction of pain by improving the flow across the minor papilla with reduction of the intraductal pressure (38). This can be achieved through minor papilla sphincterotomy, minor papilla sphincteroplasty, and dorsal duct stenting alone or in combination with sphincterotomy or -plasty (45).

A large meta-analysis demonstrated that, besides longer follow-up duration, stent implantation in the dorsal duct was the only factor associated with improved success rate of endoscopic therapy (45). Another meta-analysis indicated that the pooled overall response rate in patients with PD who received endoscopic therapy was 2/3 (36). In addition, patients with RAP-type PD were shown to respond better to endoscopic therapy than patients with chronic pancreatitis-type and pain-type PD.

A smaller case series described a 55% clinical success rate as defined by improvement of symptoms after sphincterotomy of the minor papilla and respective stent placement (46). The stents used varied in diameter from 5 to 10 Fr. As a rule, the largest possible stent was always chosen to minimize the risk of stent occlusion. The average duration of placement was 5.9 months with changes every 6 to 8 weeks.

### Alternative access

In case of an impossible transpapillary access into the pancreatic duct due to an impossible intubation, a ductal stenosis that cannot be passed or a papilla that cannot be reached, EUS-guided drainage of the pancreatic duct with stent implantation (plastic/metal) can be used as an alternative access (47). This is an efficacious method with an acceptable risk (48). Two different access routes can be differentiated: (i) transduodenal and (ii) transgastral.

Meta-analyses of several small case series show an overall clinical success rate of 78.8% and an adverse event rate of 18.9% (49). In these studies, antegrade EUS-guided puncture of the pancreatic duct through the stomach with subsequent wire advancement was performed as a first step. This was followed by dilatation of the access route and implantation of plastic stents (5-10 Fr, straight, single or double pigtail). The median length of stay was 195 days (10-780 days).

The duration of stent placement varies and must be assessed on a case-by-case basis, so that no general recommendation can be made here so far. If physiological drainage to the papilla is successful in the course of treatment, the alternative access should be removed (48).

Alternatively, EUS-guided drainage can be performed with the aid of a LAMS. So far, only one individual case report has been published (50), though the respective article concerns the drainage of a pancreatic fluid collection adjacent to the pancreatic duct rather than a dilated pancreatic duct itself.

**Figure 3:**
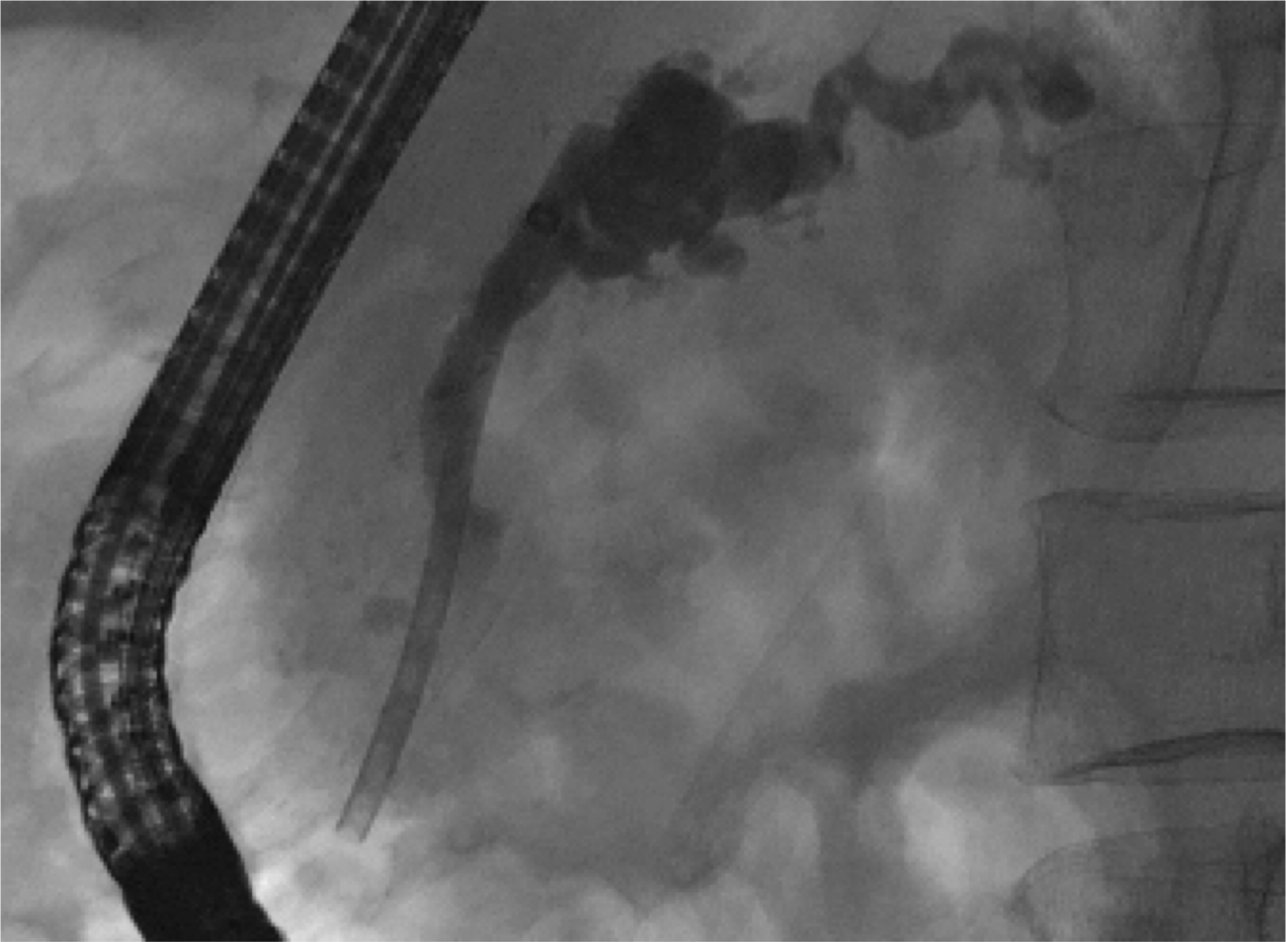
ERCP image of therapeutic pancreatic plastic stent in place in a patient with chronic pancreatitis and high grade stricture of pancreatic duct in the head. (Pictures: Ellrichmann, Conrad).

Therefore, we present our own case of successful drainage of the pancreatic duct by a LAMS:

A 70-year-old male patient was referred to our hospital end of 2021 with chronic, ethyltoxic pancreatitis and multiple pancreatic duct stones due to a stricture in the pancreatic head and respective duct dilatation up to 12 mm in diameter. Since conventional drainage *via* the papilla as well as an EUS-guided rendezvous manoeuvre failed, we decided to perform a direct gastropancreatostomy using a LAMS (HotAxios™, 8x8 mm, Boston Scientific, Malborough, MA, USA). Once established, the stones were destroyed by electrohydraulic lithotripsy *via* transgastric pancreatoscopy, all fragments were completely removed. Since the stenosis in the pancreatic head could not be passed even under pancreatoscopic guidance, the patient has an indication for a pancreatic head resection. Unfortunately, the patient still refuses any surgical therapy, therefore the stent has been left *in situ* for now approximately 10 months. Until now the clinical benefit outweighs the risk of complications such as an ingrowth of the stent (buried LAMS) or impending bleeding. However, regular endoscopic follow-up is of utmost importance. To date, the patient is completely asymptomatic (**Figure 3**).

## Technical background – available stent systems

### Plastic stents

Pancreatic plastic stents (PPSs) are primarily made of polyethylene and are available in sizes ranging from 2 to 25 cm in length and 3 to 11.5 Fr in diameter with straight, curved or single pigtail geometry.

Designated pancreatic stents have additional side holes along the entire length of the stent, which are intended to allow drainage of the side branches of the pancreas. Furthermore, PPSs exhibit one or more flaps to prevent dislocation depending on the indication and duration of use. A distinction is made between stents without an inner flap for short-term, prophylactic stenting with possible spontaneous (and intended) migration and stents with an inner flap to prevent migration for long-term use.

**Figure 4:**
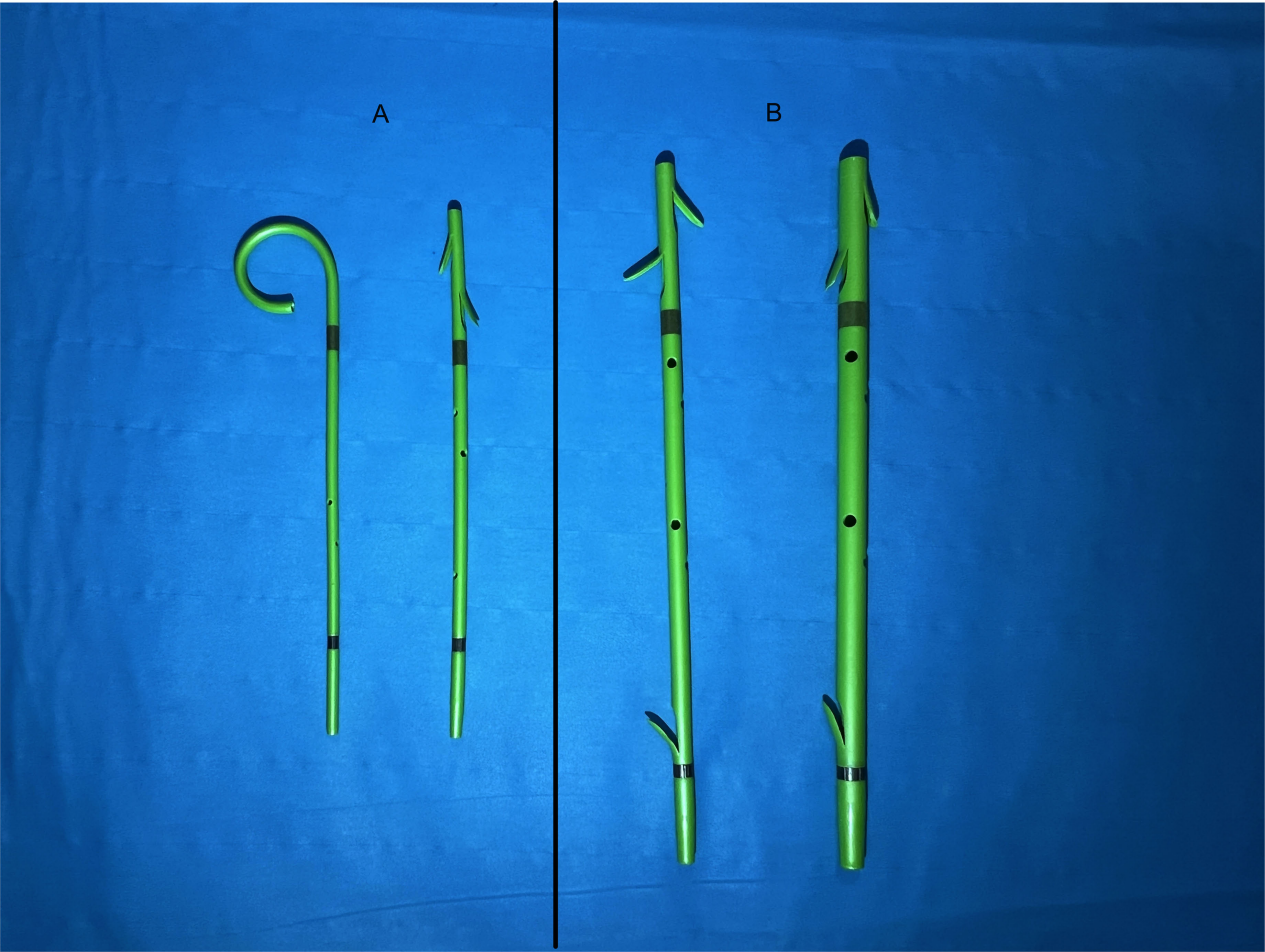
Example of prophylactic **(A)** and therapeutic **(B)** pancreatic plastic stents, Boston Scientific Advanix Pancreatic stents (Pictures: Ellrichmann, Conrad).

Technically, PPSs up to a diameter of 6 Fr are inserted using guidewire and pushing catheters only, stents with a diameter of 7 Fr and more usually come with a designated delivery system (introducer and pusher). **Table 2** gives an overview of the respective PPSs available on the market (**Table 2**; **Figure 4**).

**Table 2:** Overview of pancreatic plastic stents (PPS).

	Boston Scientific Advanix	Cook Geenen and Zimmon	Olympus Pancreatic Stent
**Length, cm**	2-18	2-20	2-12
**Diameter, Fr**	3, 4, 5, 7, 10	3, 4, 5, 6, 7, 8.5, 10, 11.5	7, 8.5, 10
**Shapes**	straight, single pigtail	single pigtail, straight	straight, s-shaped
**Flaps**	double external/double external + internal/without (pigtail only)	double external/double external + internal, without/single external	external/external + internal
**Material**	Polyethylene	Polyethylene	Polyethylene
**Indication**	prophylactic and therapeutic	prophylactic and therapeutic	therapeutic

### Self-expandable metal stents

Self-expandable metal stents (SEMSs) are primarily made of nitinol, which expands to the specified diameter when exposed to body heat within 36 hours after insertion. The architecture of the stent ensures radial expansion of the stent without sacrificing flexibility or accuracy of fit (51). The sizes of the offered stents range from 4 to 12 cm in length and from 6 to 11 mm in diameter. SEMSs can be fully covered (fc), partially covered (pc) or uncovered (uc). The coverings consist of polytetraflouroethylene, polytetraflouroethylene/flourinated ethylene propylene or silicone membranes.

fcSEMS and pcSEMS are designed to prevent ingrowth by tumor or benign tissue hyperplasia and allow easier removal and repositioning. Removal of uncovered stents is very difficult or even impossible. Therefore, they are usually used in a palliative setting (52).

The application of radiopaque stents is performed under X-ray control with the aid of additional markers (gold or titanium) at the proximal and distal end of the stent.

The SEMSs are mounted on a catheter and are covered with an outer sheath, which is retracted after placement of the stent, resulting in release of the stent. Some stents allow (to a limited extent) re-placement by re-sheath advancement up to a “point of no return” that is marked at the application system. As there are no designated SEMS for the pancreas available on the market, biliary stents are used in clinical routine (**Table 3**).

**Table 3:** Overview of self-expanding metal stents (SEMS) (in alphabetical order).

	Boston Scientific WallFlex™ Biliary RX Stent	Cook Evolution®	Microtech Biliary Stent	Olympus Hanarostent®
**Length, mm**	40-100	40-110	40-100	40-120
**Diameter, mm**	8-10	9, 11	10	6-10
**Delivery system, Fr**	8.5 (fully covered, partially covered), 8 (uncovered)	8.5	8 (uncovered), 9 (fully covered, partially covered)	5.9 (uncovered), 8.5 (fully covered)
**Covering**	fully covered, partially covered, uncovered	fully covered, partially covered, uncovered	fully covered, partially covered, uncovered	fully covered, uncovered
**Material**	Platinol	Nitinol	Nitinol	Nitinol
**Indication**	Therapeutic	Therapeutic	Therapeutic	Therapeutic

### Biodegradable stents

Biodegradable stents (BDSs) are made of polymers (e.g. polydioxanone fibers) that have so far been used in surgical sutures and degrade over time by hydrolysis (31). The geometry is based on an uncovered SEMS (ELLA-CS, Hradec Králové, Czech Republic) or has a helical design based on a regular pancreatic stent shape but without an inner lumen (AMG International GmbH, Winsen, Germany), so that the pancreatic fluids can drain at the outer surface of the device.

ELLA-CS offered a pancreatic BDS with a length of 3 or 4 cm a diameter of 6 mm, which is no longer produced. As an alternative, biliary BDS can currently be used, which are available with a diameter of 8 or 10 mm. Before application, the stent needs to be manually loaded onto the application device, thereafter, release is technically performed analogously to SEMS by retracting the outer sheath under X-ray control. With the help of titanium markers at the proximal and distal end of the stent, the position is controlled.

The biodegradable stent from AMG is offered with a length of 40 to 225 mm and a diameter of 2 to 3.4 mm. The stent is available in three different degrading profiles: (i) fast degrading within 12 days i.e. for prevention of post-ERCP pancreatitis, (ii) medium degrading within 20 days and (iii) slow degrading within 11 weeks i.e. for pancreatic duct strictures. **Table 4** gives an overview of the available BDS.

**Table 4:** Overview of biodegradable stents.

	Ella-CS DV Stent Biliary THP	AMG International GmbH Archimedes Stent
**Length, cm**	3-8	4-12,5
**Diameter, mm**	8, 10	2, 2.6, 3.4
**Delivery System, Fr**	11.5	11.5
**Material**	Polydioxanone	No information available
**Features**	–	Fast (12 days), medium (20 days) and slow (11 weeks) degradation profile

### Lumen apposing self-expandable metal stents

Lumen apposing self-expandable metal stents (LAMSs) are a subgroup of SEMS with a special, barbell- like geometry, thereby ensuring the apposition of two lumina to each other and minimizing the risk of subsequent leakage or perforation (53). They are integrated into a single-step platform and are used under endoscopic-ultrasound (EUS) guidance for decompression of obstructed ducts, among other applications. The stents are available with a saddle of 8 to 15 mm and an inner diameter of 6 to 20 mm, though diameters of 6 to 8 mm should be preferred in pancreatic duct drainage. The offered systems combine puncture and stent application so that no device change is necessary.

After electrocautery-assisted puncture, the stent is released in several predefined steps. **Table 5** gives a brief overview of the most common stents available.

**Table 5:** Overview of lumen-apposing metal stents (LAMS) with electrocautry tip (in alphabetical order).

	Boston Scientific Hot Axios™ Stent	TaeWoong Niti-S™ HOT SPAXUS™ Stent
**Saddle length, mm**	8, 10, 15	20
**Diameter, mm**	6, 8, 10, 15, 20	8, 10, 16
**Delivery system, Fr**	10	10
**Material**	Nitinol	Nitinol

## Conclusion

Stenting of the pancreas is a challenging task for the interventional gastroenterologist. The main indications for pancreatic stent implantation are either prophylactic or therapeutic.

Prophylactic stent implantation aims to prevent PEP by using thin (3-5 Fr) and short (3-5 cm) designated pancreatic stents at least in high-risk patients.

Therapeutic stent placement aims to restore the proper flow of pancreatic secretion with stenoses, leaks, fistulas or anatomical malformation of the pancreatic duct. Depending on the etiology, plastic stents or SEMSs are used.

Another field of pancreatic stenting represents EUS-guided puncture with stent implantation as an alternative access to the main pancreatic duct when transpapillary access is impossible. In addition to the implantation of plastic stents, which achieve good results, LAMS implantation can be an alternative method with promising results.

To sum up, the field of pancreatic stenting is complex and belongs in the hands of experienced endoscopists in specialized institutions. This can ensure that the patient receives the optimal treatment with the best possible outcome.

## Data availability statement

The original contributions presented in the study are included in the article/Supplementary Material. Further inquiries can be directed to the corresponding author.

## Author contributions

ME: Literature search, preparation of review and manuscript, conduction of endoscopies. CC: Literature search, preparation of review and manuscript, conduction of endoscopies. All authors contributed to the article and approved the submitted version.
